# P-702. Pathogen Detection Among Hospitalized Adult Patients with and without Severe Community-Acquired Pneumonia

**DOI:** 10.1093/ofid/ofaf695.914

**Published:** 2026-01-11

**Authors:** Ramara E Walker, Michael Rothberg, Abhishek Deshpande, Andrea Pallotta, Rebecca Schulte, Ming Wang

**Affiliations:** Cleveland Clinic, Cleveland, OH; Cleveland Clinic, Cleveland, OH; Cleveland Clinic, Cleveland, OH; Cleveland Clinic, Cleveland, OH; Cleveland Clinic, Cleveland, OH; Case Western Reserve University, Cleveland, Ohio

## Abstract

**Background:**

Community-acquired pneumonia (CAP) is a leading cause of hospitalization and mortality in the US. Viruses have an increasing role in the etiology of CAP. The etiology of CAP in the post-Coronavirus Disease (COVID) era is unknown.

**Figure 1.**
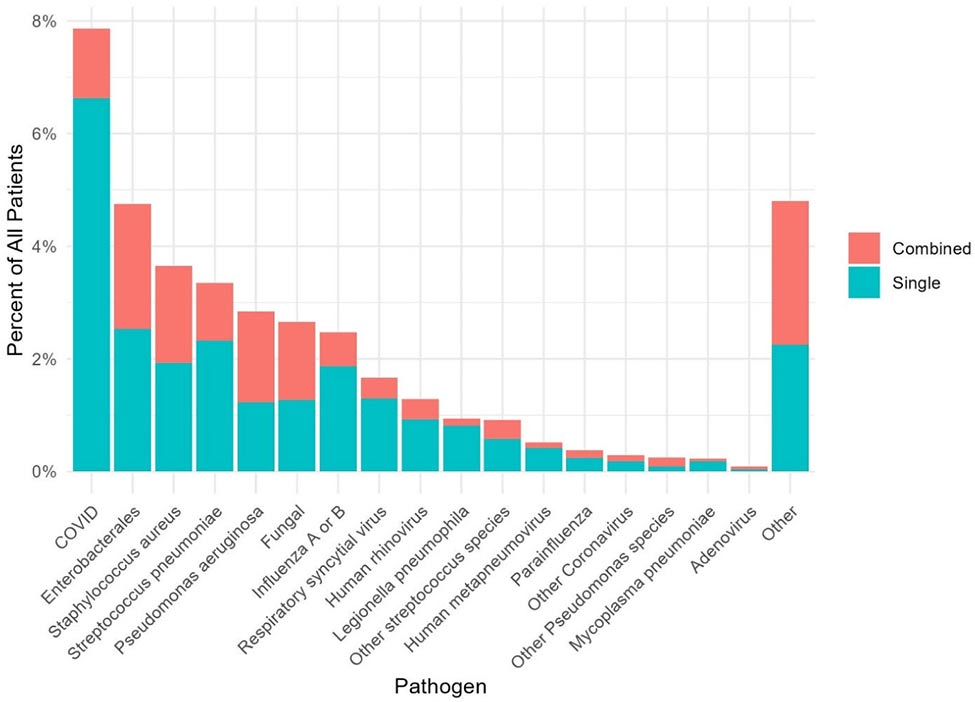
Pathogen Detection among Hospitalized Adults with Community-Acquired Pneumonia, 2022-2025.

**Table 1.**
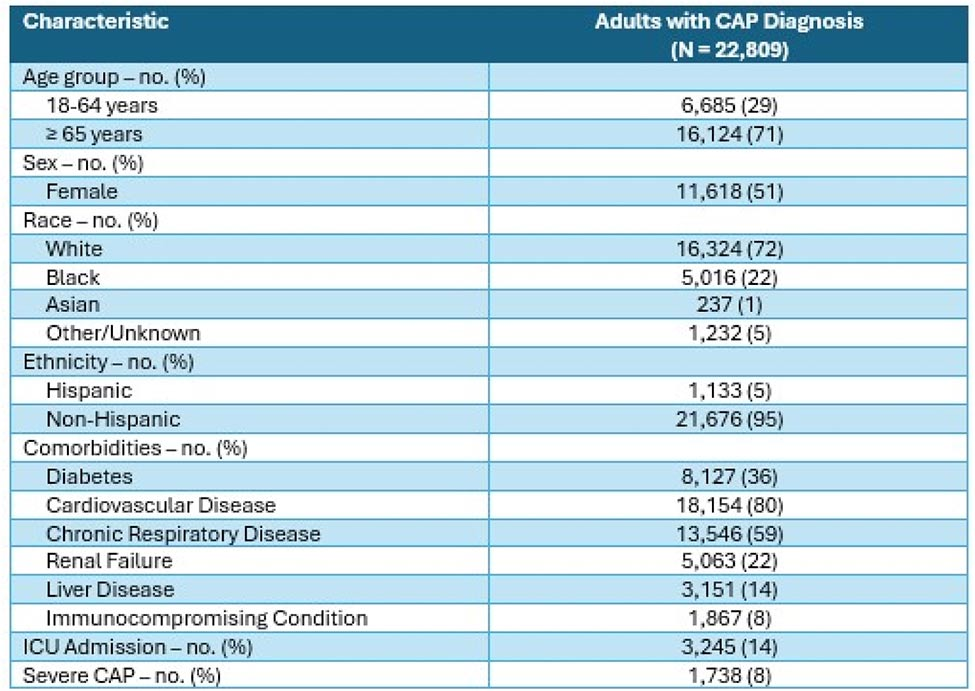
Characteristics of Adult Patients Hospitalized with Community-Acquired Pneumonia

**Methods:**

This was a retrospective ecological study of patients ≥ 18 years hospitalized with CAP at 12 Cleveland Clinic hospitals between November 1, 2022 – February 28, 2025. We reviewed blood, urine, and respiratory specimens collected for culture, antigen detection, and molecular diagnostic testing conducted during routine care. We compared patients with and without severe CAP using descriptive statistics.

**Table 2.**
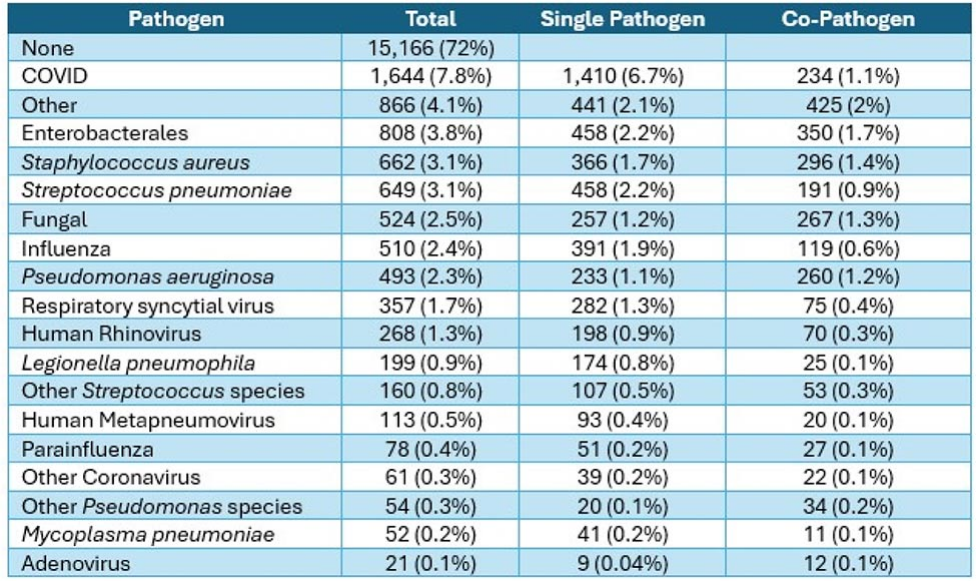
Specific Pathogen Detection in Patients with Non-Severe Community-Acquired Pneumonia

**Table 3.**
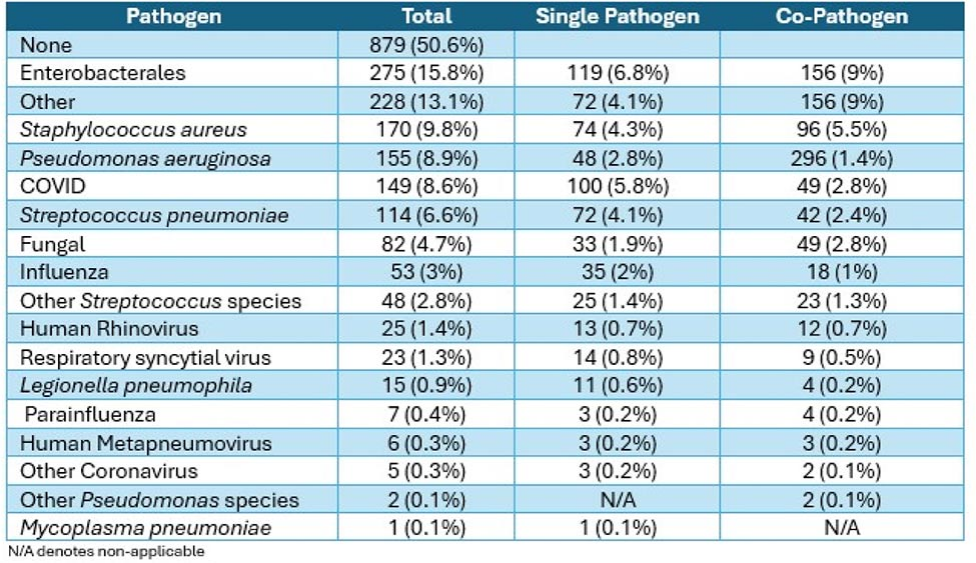
Specific Pathogen Detection in Patients with Severe Community-Acquired Pneumonia

**Results:**

Among 22,809 patients admitted with CAP, most were ≥ 65 years (71%), 11,618 were female (51%), 16,324 were white (72%), and cardiovascular disease was the most frequent comorbidity (80%); 3,245 (14%) were admitted to the intensive care unit and 1,738 (8%) had severe CAP (Table 1). Compared to patients with non-severe CAP, those with severe CAP were more likely to have blood cultures (90% vs. 65%), respiratory culture (43% vs. 27%), and expanded respiratory panel (18% vs. 14%), but not testing for influenza (85% vs. 86%), COVID (89% vs. 91%), respiratory syncytial virus (84% vs. 86%), or urinary antigen testing (UAT) for *S. pneumoniae* (56% vs. 61%) and *L. pneumophila* (53% vs. 51%). A pathogen was detected in 6,764 (30%) patients overall (Figure 1); 859 in patients with severe CAP (49%) and 5,905 in non-severe (28%). Among those that had a pathogen detected, the composition of pathogens isolated in those with and without severe CAP was bacteria alone (45% vs. 34%), one or more viruses (28% vs. 49%), bacterial and viral (21% vs. 9.6%), and fungal or mycobacterial (3.4% vs 3.9%). The most common pathogens isolated in non-severe CAP were COVID (7.8%), Enterobacterales (3.8%), and *S. aureus* (3.1%) (Table 2). The most common pathogens isolated in patients with severe CAP were Enterobacterales (15.8%), *S. aureus* (9.8%), and *P. aeruginosa* (8.9%) (Table 3).

**Conclusion:**

Among patients with CAP, most did not have a causative organism identified. When one was identified, severe CAP was most commonly caused by bacterial pathogens; non-severe CAP was most often due to COVID.

**Disclosures:**

All Authors: No reported disclosures

